# A Nanoconfined Four-Enzyme Cascade Simultaneously Driven by Electrical and Chemical Energy, with Built-in Rapid, Confocal Recycling of NADP(H) and ATP

**DOI:** 10.1021/acscatal.2c00999

**Published:** 2022-07-08

**Authors:** Clare F. Megarity, Thomas R. I. Weald, Rachel S. Heath, Nicholas J. Turner, Fraser A. Armstrong

**Affiliations:** † Department of Chemistry, University of Oxford, South Parks Road, Oxford OX1 3QR, U.K.; ‡ School of Chemistry, Manchester Institute of Biotechnology, University of Manchester, 131 Princess Street, Manchester M1 7DN, U.K.

**Keywords:** protein film electrochemistry, enzyme cascades, nanoconfinement, cofactor recycling, kinases, biocatalysis

## Abstract

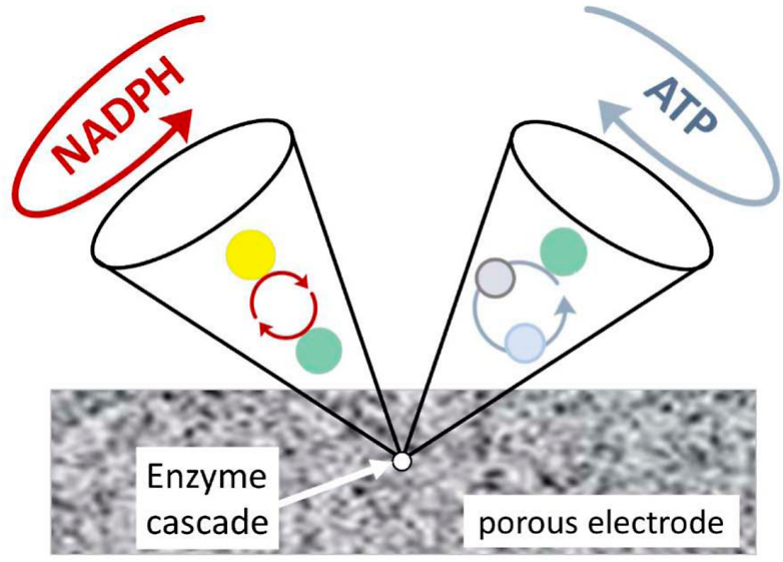

The importance of energized nanoconfinement for facilitating the study and execution of enzyme cascades that feature multiple exchangeable cofactors is demonstrated by experiments with carboxylic acid reductase (CAR), an enzyme that requires both NADPH and ATP during a single catalytic cycle. Conversion of cinnamic acid to cinnamaldehyde by a package of four enzymes loaded into and trapped in the random nanopores of an indium tin oxide (ITO) electrode is driven and monitored through the simultaneous delivery of electrical and chemical energy. The electrical energy is transduced by ferredoxin NADP^+^ reductase, which undergoes rapid, direct electron exchange with ITO and regenerates NADP(H). The chemical energy provided by phosphoenolpyruvate, a fuel contained in the bulk solution, is cotransduced by adenylate kinase and pyruvate kinase, which efficiently convert the AMP product back into ATP that is required for the next cycle. The use of the two-kinase system allows the recycling process to be dissected to evaluate the separate roles of AMP removal and ATP supply during presteady-state and steady-state catalysis.

## Introduction

Many of the complex chemical pathways of living cells involve catalysis by enzyme cascades confined in zones such as the mitochondrion or Golgi body.^1,2^ Enzyme crowding and substrate channeling, resulting from this confinement, are two reasons proposed to account for high rates, efficiency, and selectivity.^3−7^ Considering that total macromolecule levels are typically 400 g L^–1^ in mitochondria and 200 g L^–1^ in the cytosol of a eukaryotic cell,^8−10^ it seems certain that enzyme concentrations in vivo far exceed the high dilutions applying in conventional steady-state enzyme kinetic studies; however, the cellular concentration of any single enzyme is much less important than the systematic corralling of different interdependent enzymes. There is an increasing interest in mimicking nature’s nanoconfinement in vitro for enhanced cascade biocatalysis.^3,4,6,11^ Strategies fall under two approaches: (i) surface-confined, in which enzymes are tethered to a surface,^12−15^ and (ii) volume-confined, in which the enzymes are encapsulated in compartments such as microdroplets^16−18^ or protein cage-like structures,^19,20^ incorporated within the matrix of a metal-organic framework^21,22^ or immobilized within a hydrogel.^23^ The various efforts focus on the synthesis of often intricate enzyme nanoconfinement platforms and measurements of their performance.

**Figure 1:**
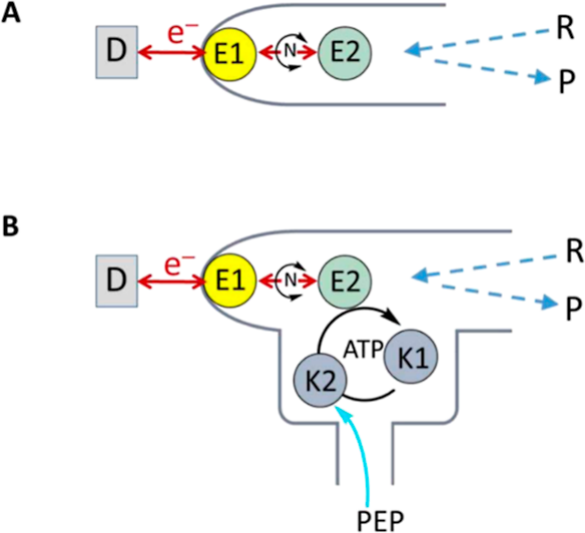
Maps of enzyme cascades energized, observed, and controlled under nanoconfinement. D represents dashboard control. (A) Basic cascade with E1 = FNR, N = NAD(P)(H), E2 = dehydrogenase. (B) Incorporation of kinases K1 and K2 to achieve confocal ATP recycling using phosphoenolpyruvate (PEP) as fuel.

We recently discovered that enzyme cascades can be confined, driven, controlled, and monitored (in “real-time”) within the nanopores formed naturally when indium tin oxide (ITO) nanoparticles are deposited on a conducting support.^24−33^ ITO (typical composition <78% In, >4% Sn) is electronically conductive. The basic concept is illustrated as a map in Figure 1A. If one of the entrapped enzymes (E1) is able to catalyze the direct electrochemical interconversion of nicotinamide cofactors, a new way emerges for exploiting enzyme cascades and investigating enzyme reactions.

One such enzyme, ferredoxin NADP^+^ reductase (FNR), exhibits rapid and reversible direct electron exchange between its active site flavin adenine dinucleotide (FAD) cofactor and the ITO material at which it binds tightly, enabling it to recycle NADP(H) locally to drive the reactions of a neighboring dehydrogenase (E2).^24,26,27^ In green plants, FNR is responsible for channeling the energetic electrons generated by photosynthesis into organic chemistry: accordingly, the nanoporous electrode loaded with different enzymes and known as the Electrochemical Leaf (e-Leaf) is able to mimic practical traits of catalysis in living cells.^24,26,27,31^ Nanoconfinement of enzyme cascades in such a way offers important advantages over experiments carried out on solutions: (a) in providing a high local concentration of adjacent (producer/receiver) enzyme pairs, and by extension, teams of enzymes; (b) in promoting the local retention of intermediates and exchangeable cofactors [such as NAD(P)(H)] so that they are processed or recycled before they diffuse away; (c) in enabling the power of dynamic electrochemical methods to energize, control, and observe the processes in a highly interactive way. An enticing and useful analogy is that the experiments are now run from a “dashboard” D, which includes the electrochemical workstation (direction, driving force, rate, and progress) and the means to add or remove reagents to/from the immobilized catalysts.

The rapid, simple, and inexpensive electrophoretic deposition of ITO nanoparticles on a conductive support such as graphite or titanium foil results in a robust layer 1–3 μm thick, depending on deposition time (2–10 min), rich in pores less than 100 nm in diameter into which enzymes permeate.^24,27,34^ The procedure creates random nanospace to confine enzyme cascades that can now be energized and observed electrochemically through the action of FNR. Molecules of FNR (MW 32 kDa) bind with high affinity and are visible and quantifiable through two-electron reversible cyclic voltammetry due to the FAD cofactor, the reduction potential being as expected for the free enzyme measured in solution.^24,27^ The electroactive coverage at pH 7.5 equates to many tens of monolayers, and estimations based on a 1 μm penetration depth show that its local concentration may approach 1 mM.

The map shown in Figure 1A is the minimal cascade unit - an enzyme pair consisting of FNR (E1) and a dehydrogenase (E2), each of which is tightly bound in the nanopores, along with a molecule of NADP(H) that exchanges between the two.^26^ Examples of E2 so far include glutamate dehydrogenase, native and variant isocitrate dehydrogenases, and alcohol dehydrogenases.^26−28,30,32,33^ By its tight coupling to E1 via localized and bidirectional NADP(H) recycling (E1 ↔ hydride ↔ E2), E2 is itself rendered electroactive. Accordingly, the e-Leaf now extends protein film electrochemistry (PFE), which has provided unique insight into the properties of enzymes that use long-range electron transfer,^35−38^ to the investigation of a class that includes 1/10^th^ of all enzymes. The electrode is thus described by the notation (E1 + E2 + ...)@ITO/support, where E1 acts as a transducer, translating the rate of chemical flow into electrical current. An extended linear cascade (in which E1 = FNR, E2 = l-malate NADP^+^-oxidoreductase, E3 = fumarase, and E4 = l-aspartate ammonia lyase) has been used to perform the electrocatalytic synthesis of l-aspartate from pyruvate, CO_2_, and NH_4_^+^: inclusion of carbonic anhydrase (E2A—comprising a branch linked to E2) allowed bicarbonate (HOCO_2_^–^) to be used in place of CO_2_.^31^ The three nonredox enzymes in the linear chain could be driven in either direction, synthesis or oxidation of aspartate, simply by varying the electrode potential, while the rate and progress of the overall reaction were monitored as current and accumulated charge. Such confinement of all enzymes in an inexpensive electrode material, along with NADP(H), which is required only at low levels, lends itself to scaleable electrochemical reactors.^30^

The other major exchangeable cofactor for biocatalysis is ATP. We thus questioned if NADP(H) and ATP recycling might be coupled and engaged in a confocal manner, whereby the recycling of both cofactors is required to occur in the same region. To gain a mechanistic insight into how this advantage can be achieved, we sought an enzyme requiring both NADPH and ATP and constructed the cascade depicted in Figure 1B, in which a two-kinase recycling system is co-entrapped.

**Scheme 1:**
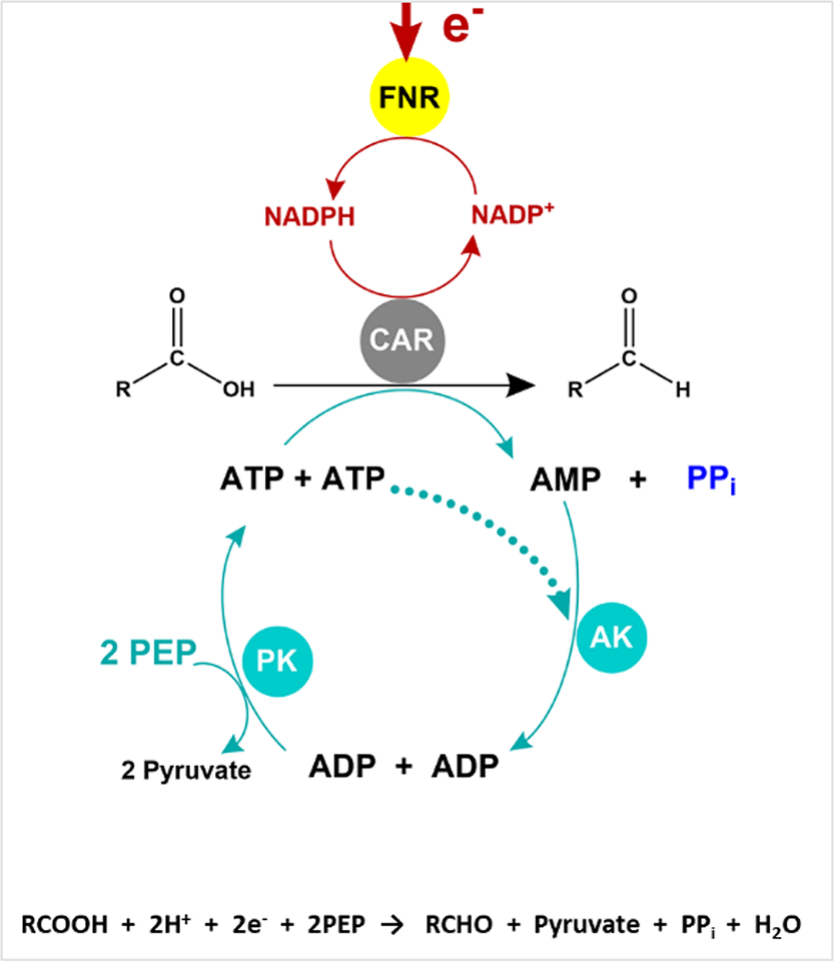
Flow Chart Showing Confocal Recycling of NADP(H) and ATP by a Nanoconfined Cascade in the Electrochemical Leaf (e-Leaf) Electrical energy supplied to the porous ITO electrode is transduced by entrapped FNR to regenerate NADP(H) (red arrows) locally; chemical energy supplied as a fuel in the form of PEP (cyan arrows) is transduced by the kinase pair, AK (E.C. 2.7.4.3), and PK (E.C. 2.7.1.40) also trapped in the pores. The central reaction, the reduction of a carboxylic acid to an aldehyde catalyzed by CAR, is simultaneously energized by both branches. The overall reaction is written below.

Carboxylic acid reductase (CAR) (EC 1.2.1.30) catalyzes the reduction of carboxylic acids to their respective aldehydes, a reaction that consumes NADPH and ATP (which is converted to AMP). The enzyme from *Segniliparus rugosus* has been characterized by X-ray crystallography: it has two mobile domains, the N-terminal domain containing the adenylation site and the C-terminal domain housing the reductase.^39−41^ In the proposed mechanism, ATP binds with the carboxylic acid substrate and a bound phosphoester intermediate is formed with the release of pyrophosphate (PP_i_): in the subsequent step, a thioester intermediate is formed, and AMP is released. The arm of the enzyme then flips to the reductase domain, where the thioester is reduced by NADPH to give the aldehyde product.^41^ The crystal structure reveals a molecule of AMP, presumably bound tightly in the state of the enzyme that is purified. A recent paper described how catalysis by CAR could be carried out in solution, using a glucose dehydrogenase to regenerate NADPH and polyphosphate kinase to regenerate ATP.^42^ The enzyme offered an ideal subject with which to examine how electrochemically driven NADP(H) recycling and chemically driven ATP recycling can be combined simultaneously under the condition of nanoconfinement. The resulting system (Figure 1B) incorporated adenylate kinase (AK = K1) to convert AMP and low-level ATP to ADP and pyruvate kinase (PK = K2) to convert ADP back to ATP using PEP as a small chemical fuel molecule. The primary enzyme cascade, consisting of FNR and CAR, is thus linked to a service branch that recycles ATP. The recycling system operates very locally and offers insights into the importance of two *separate* tasks: (a) providing ATP to CAR and (b) assisting in the removal of AMP from CAR (Scheme 1).

Table 1 lists sizes and kinetic characteristics of the enzymes used. Of the four enzymes, only FNR can be quantified on the electrode through the size of the prominent two-electron voltammetric peaks due to FAD; quantities of each of the other enzymes present on the electrode are varied by adjusting the loading ratio. Notably, CAR is an inherently slow enzyme and the one most likely to limit the rate (current), whereas the kinases are very active. For FNR, the turnover frequency for NADP^+^/NADPH interconversion correlates with the rate of direct electron tunneling between the ITO surface and the FAD active site, which depends strongly on the electrode potential that is applied. Two types of electrochemical experiment were used to monitor electrocatalysis, cyclic voltammetry to study potential and waveshape, and chronoamperometry to conduct cinnamaldehyde electrosynthesis and reveal the effect of periodic refueling with PEP.

**Table 1:**
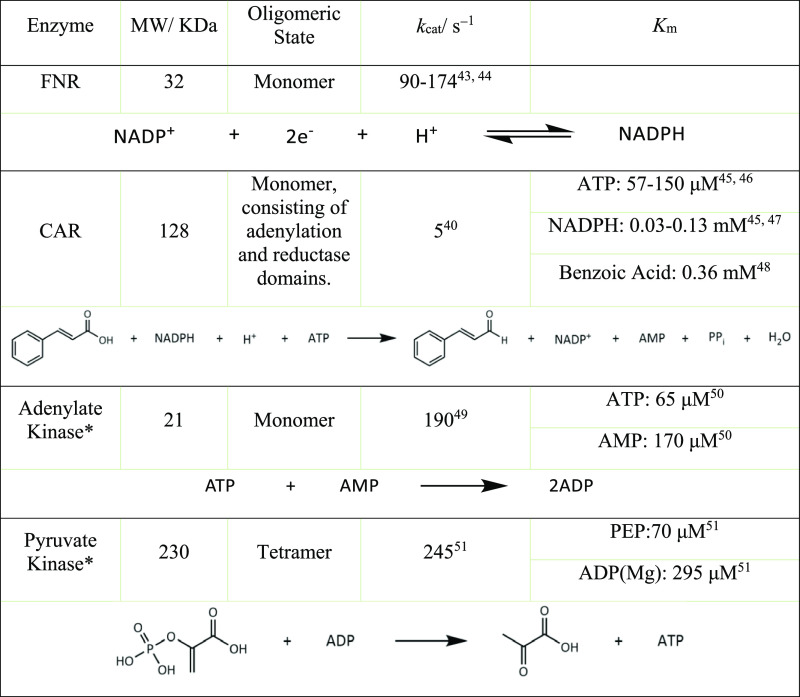
Parameters for the Enzymes Used in This Investigation, with References Giving Further Information

a Commercially prepared from rabbit muscle (Merck (Sigma)).

As with PFE applied to electron-transport enzymes, it was expected that the shapes of the electrocatalytic voltammograms obtained with the e-Leaf under different times and conditions would provide important basic insights into the operation of the cascade and factors limiting catalysis. Three scenarios were anticipated.

(1) *Control by FNR (electron transfer and NADP^+^reduction)*. The current would have a strong and persistent potential dependence showing that interfacial electron transfer is rate limiting. Since FNR displays fast electron exchange with the ITO surface and rapid, quasireversible NADP(H) recycling when studied in isolation, this condition would apply only if turnover by E2 is sufficiently fast to produce a high demand on the rate of NADPH recycling.
(2) *Steady-state catalysis without an intermediate limiting the rate*. A sigmoidal wave would be obtained showing a current plateau: this result would be observed if the reaction rate is limited by reactions occurring at E2 or other enzymes.
(3) *An intermediate is depleted*. A peak-like current response would indicate that an essential reactant is being depleted (consumed) faster than it can be replaced.

## Methods

**Figure 2:**
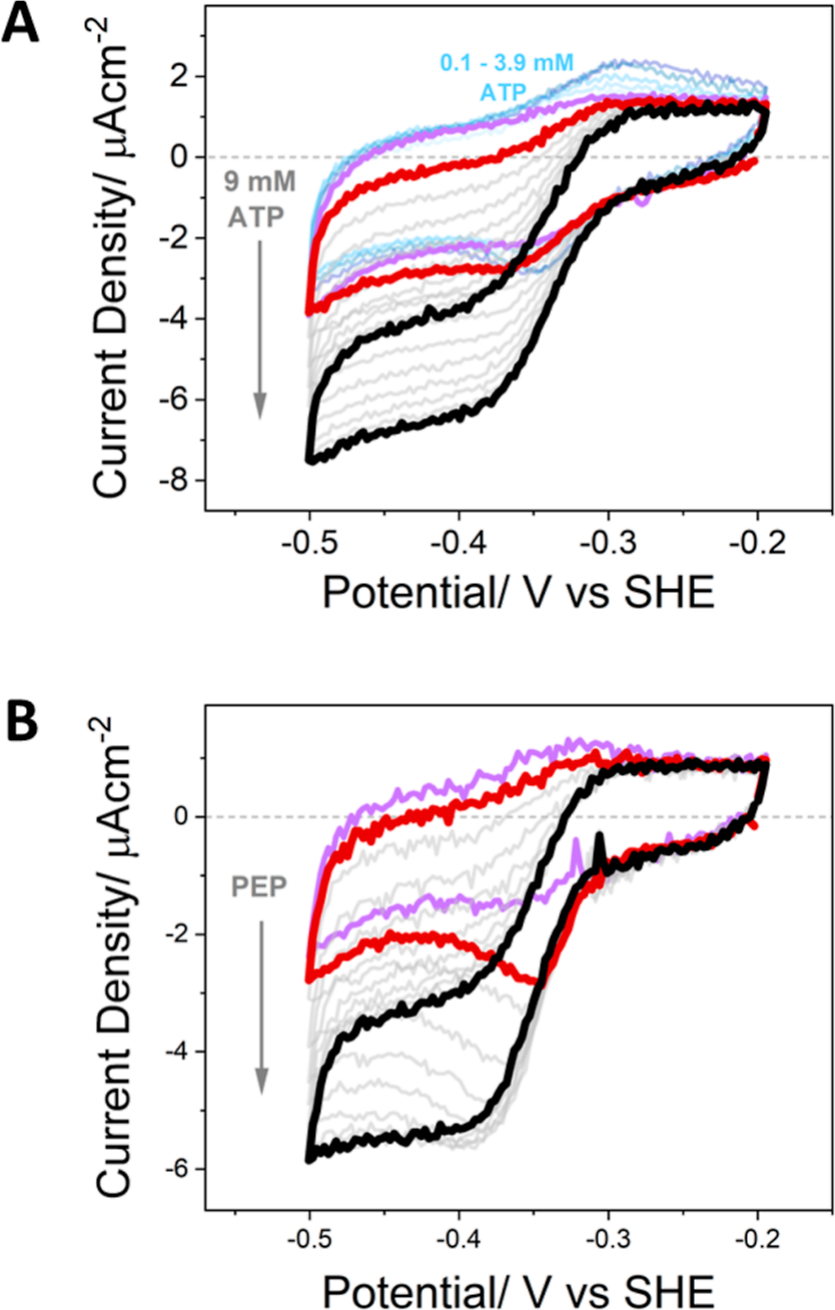
(A) Reduction of cinnamic acid by CAR coupled to the interconversion of NADP^+^/NADPH by FNR as monitored by cyclic voltammetry at a scan rate of 1 mV s^–1^ (each cycle taking 10 min). The violet scan corresponds to the background activity of the NADP^+^/NADPH interconversion by FNR. The coupled reaction was initiated by the addition of ATP titrated from 0.1 to 9 mM [initial scans corresponding to the lower ATP range of 0.1–3.9 mM shown in blue, and intermediate pale gray scans graduating to the final black scan correspond to the growth in current upon addition of ATP to give a total concentration of 9.0 mM (the first scan after this addition is highlighted in red)]. All other reactants present from the start: 20 μM NADP^+^ (in a standard reaction buffer, Supporting Information 1.2.3). Electrode surface area: 4 cm^2^ (1 double-sided Ti foil) stationary electrode; 25 °C. (B) In situ generation and recycling of ATP driving the four-enzyme cascade when primed with 10 μM ATP; cascade activity initiated by the addition of PEP to a final concentration of 2.5 mM, and all other substrates and cofactors present from the start (20 μM NADP^+^ and 1 mM AMP in standard reaction buffer; the kinase ratio was 10 PK: 1 AK). Electrode surface area: 6 cm^2^ (1 double-sided Ti foil); scan rate 1 mV s^–1^; stationary electrode; 25 °C.

**Figure 3:**
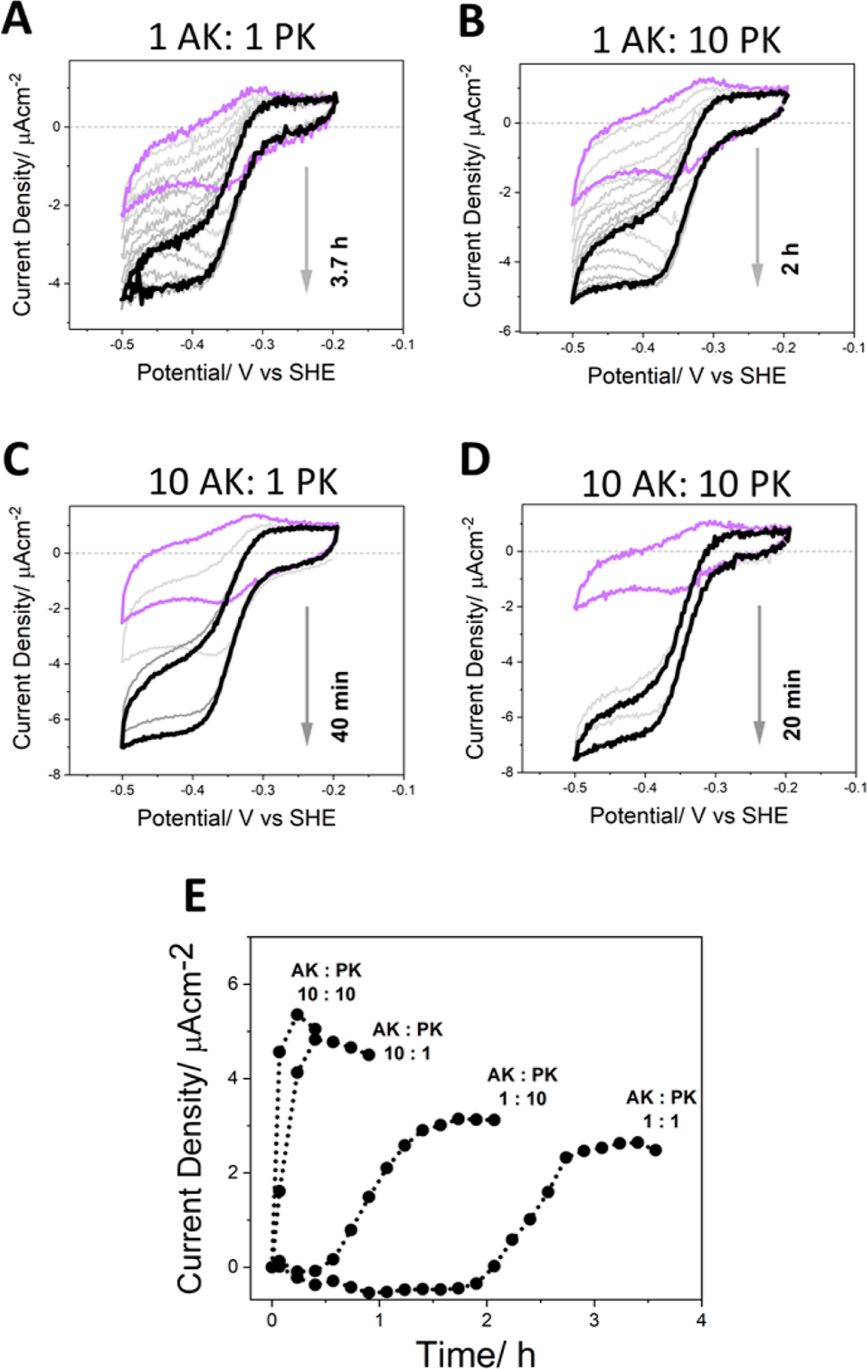
(A–D) Cyclic voltammograms (scan rate 1 mV s^–1^) showing the development of the electrocatalytic activity of the complete four-enzyme cascade shown in Scheme 1 under four different kinase (AK/PK) ratios. The violet scan corresponds to the background activity of the NADP^+^/NADPH interconversion by FNR. Reactions were initiated by adding PEP to a final concentration of 2.5 mM (pale gray scans graduating to black correspond to the growth in coupled current after this addition); all other substrates and cofactors present from the start (20 μM NADP^+^ and 1 mM AMP in the standard reaction buffer (Supporting Information 1.2.3). Kinase ratios were calculated as the number of mols based on tetrameric PK and monomeric AK, where 1 represents 0.04 nmols of enzyme in the loading solution, and 10 represents 0.4 nmols. The electrode surface area for each experiment was 5.8 cm^2^ (1 double-sided Ti foil). Other conditions: electrode stationary, 25 °C. (E) The time dependences of the increase in electrocatalytic current density for each of the kinase ratios shown in (A–D). The current density was measured at −0.45 V vs SHE during the reductive sweep of the cyclic voltammograms and plotted against the corresponding time since the initiation of the coupled reaction.

**Figure 4:**
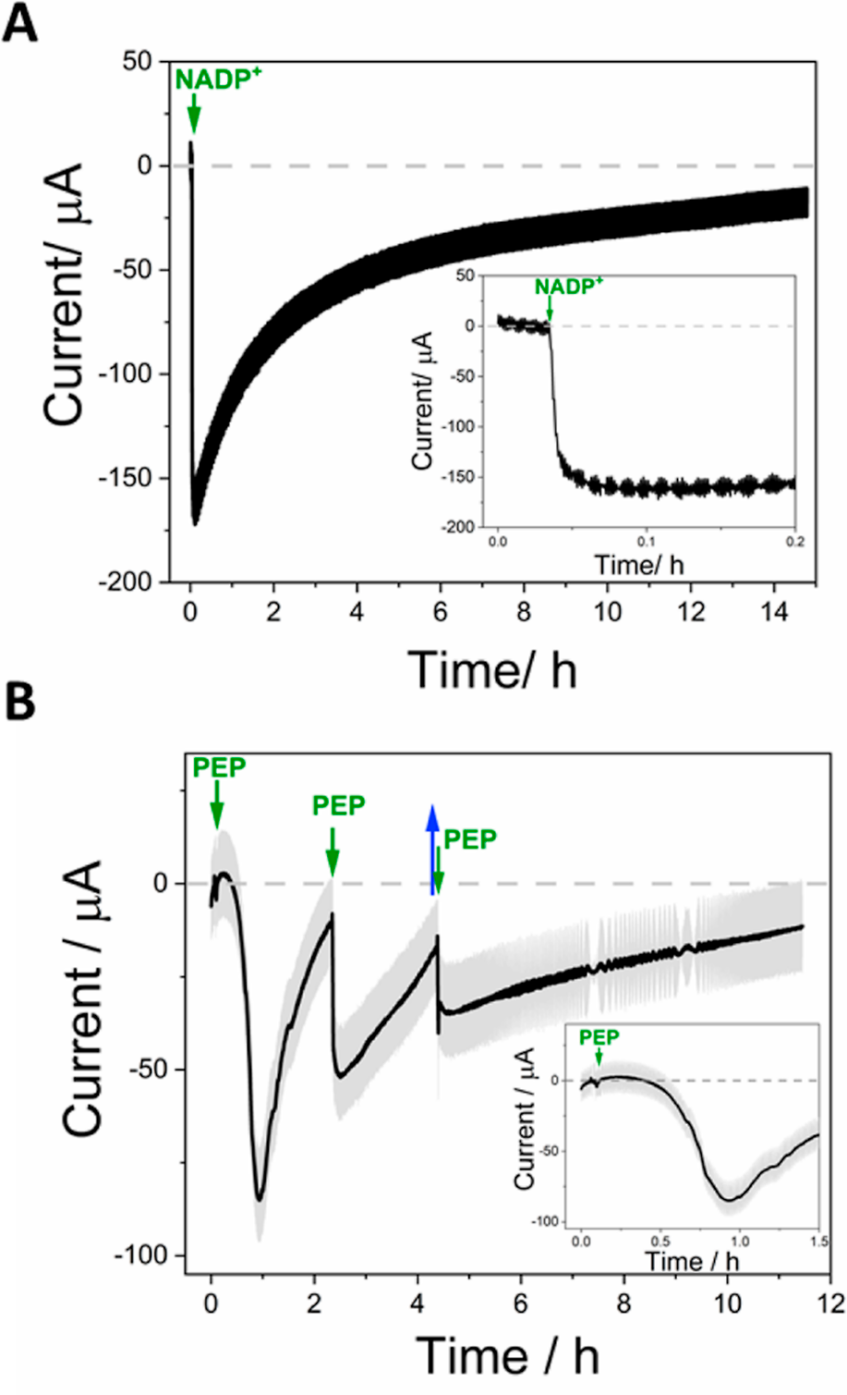
Production of cinnamaldehyde by CAR driven via FNR and NADP(H) and by the in situ generation of ATP by PEP catalyzed by AK and PK. Potential held at −0.43 V vs SHE to drive the reduction; experiments performed in an anaerobic glovebox to avoid any contribution to the current from the reduction of O_2_. (A) Cascade initiated by the addition of NADP^+^ to a final concentration of 20 μM; all reactants present from the start (10 mM cinnamic acid, 5 mM AMP, 5 mM PEP, 10 mM MgCl_2_, 10 mM KCl, 5 mM NaP_i_, in 100 mM HEPES, pH 7.5); solution stirred continuously throughout; inset shows a magnification of the live injection of NADP^+^; electrode surface area: 25 cm^2^ (a booklet of 5 double-sided Ti foils), (B) Cascade initiated by adding PEP to a final concentration of 1 mM (all other reactants present from the start: 20 μM NADP^+^, 1 mM AMP, 10 mM cinnamic acid 10 mM MgCl_2_, 10 mM KCl, 5 mM KP_i_, in 100 mM HEPES, pH 7.5) and refueled by two further additions of PEP (green arrows). Blue arrow indicates the removal of a sample for NMR analysis. Electrode surface area: 28 cm^2^ (a booklet of 5 double-sided Ti foils). Other conditions: 25 °C, solution stirred throughout.

**Figure 5:**
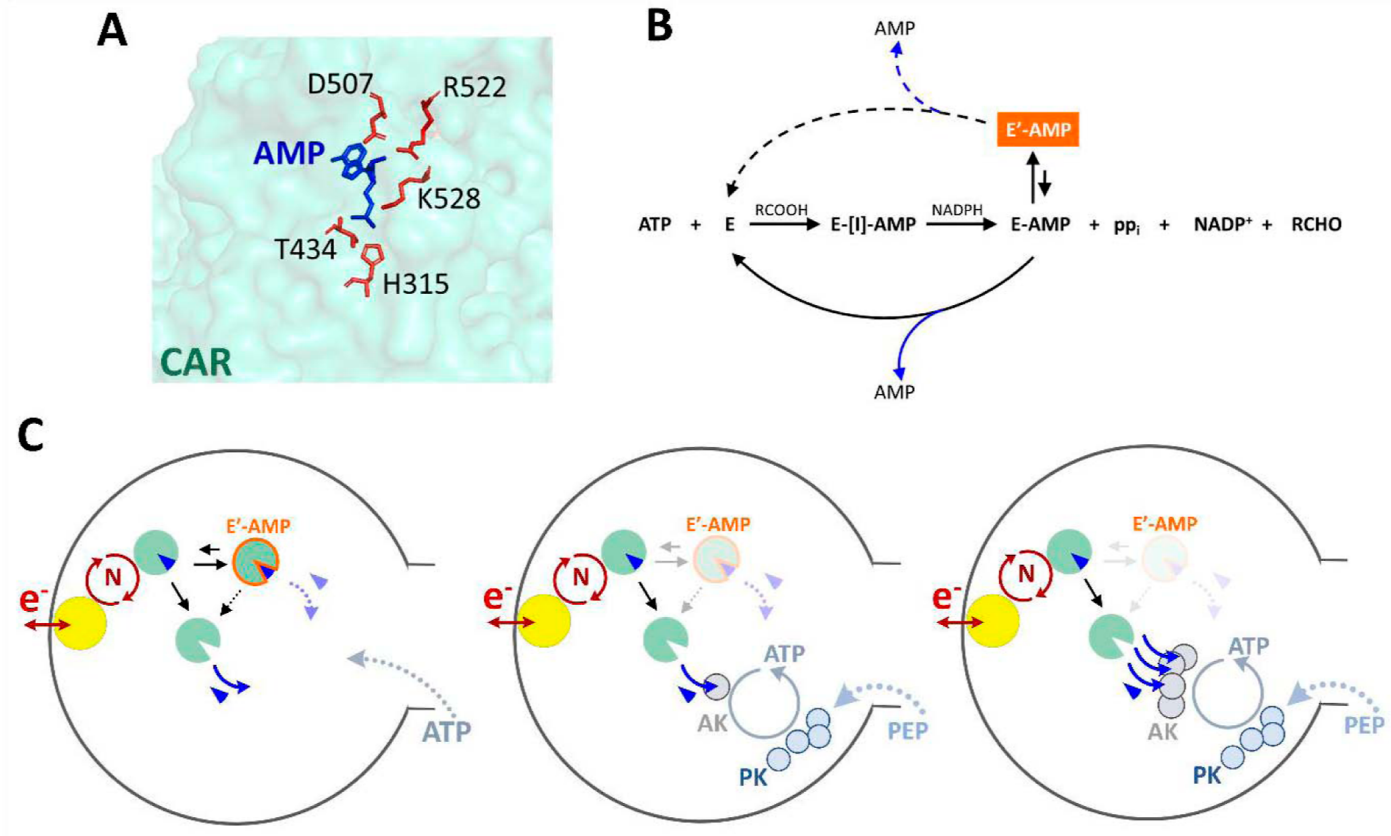
Proposal for how the capture of the tightly bound AMP product, revealed in the crystal structure of CAR (A), accounts for the efficiency of confocal ATP accumulation and recycling in situ compared to ATP supplied from the bulk solution. (B) Possible outcomes throughout the catalytic cycle of CAR: NADPH binds to the enzyme species with the carboxylic acid intermediate and AMP both bound; the reduction step is then catalyzed to produce the aldehyde. At this point, E-AMP either dissociates, releasing AMP to regenerate active CAR, or reverts to a “resting inactive state” shown in orange, in which AMP is more tightly bound (which is the state revealed in the crystal structure). (C) Nanoconfined system without the kinase cascade (left) and with the kinase cascade with a constant amount of PK but different levels of AK (middle and right). The special importance of AK lies in its ability to sequester the AMP (blue solid arrows), allowing CAR to start the next cycle. Without the kinase cascade present, the probability that the E’AMP state persists in the pores is high since AMP sequestration is not possible; hence it is shown in bold. With the kinase cascade present, E′-AMP is less persistent [shown by increasing transparency as the level of AK increases (middle to right)]; thus, the system with more AK achieves an optimal steady state more rapidly than the system with less AK. The lag period (Figure 3E) is thus determined not only by how rapidly the ATP level increases from its trace level but also by how quickly active CAR is regenerated from the inactive E’AMP complex aided by the removal of local AMP by AK.

All electrochemical experiments were performed under anaerobic conditions in a glovebox (MBraun or Belle Technologies) containing a N_2_ atmosphere (O_2_ < 2 ppm), with a three-electrode configuration using a three-compartment glass cell for chronoamperometry or a two-compartment cell for cyclic voltammetry. Electrochemical measurements were made using an Autolab (PGSTAT128N) or EcoChemie potentiostat with Nova software (full details in the Supporting Information 1.2.2). Potentials € are quoted with respect to the standard hydrogen electrode (SHE) using the correction *E*_SHE_ = *E*_SCE_ + 0.241 V at 25 °C or *E*_SHE_ = *E*_Ag/AgCl_ + 0.21 V.^52^ Electrodes were prepared by electrophoretic deposition of ITO nanoparticles onto each side of pieces of Ti foil, as described previously^24^ (and detailed in the Supporting Information 1.2.1). FNR from *Chlamydomonas reinhardtii* was expressed in *Escherichia coli* and purified as described previously^26,30^ (see also the Supporting Information 1.1). Post-translational phosphopantetheinylation of CAR is required for maximum enzyme activity; therefore, CAR from *S. rugosus* was co-expressed in *E. coli* with a phosphopantetheine transferase from *Bacillus subtilis*(40) (see the Supporting Information 1.1 for details). Rabbit muscle PK (Type VII) and AK were obtained from Merck (Sigma). The enzymes, FNR, CAR and, when included, AK and PK, were mixed and loaded as follows: a fresh ITO@Ti electrode was placed in a buffered solution (100 mM TAPS, pH 8) containing the specified number of moles of each enzyme and stirred overnight in a cold room at 4 °C. In order to ensure that the electrode was fully submerged, the total volume of the loading solution ranged from 3 mL (for the experiments shown in Figures 2 and 3) to 8 mL (for the book of larger electrodes used in Figure 4). Before use, the electrode was rinsed thoroughly in a stream of ultrapure water (Millipore, 18 MΩ cm) to remove any unbound enzyme. This rinsing took place outside the glovebox, following which the electrode was immersed in fresh buffer (the same as that intended to be used in the experiment) and purged in the glovebox port for several minutes before being taken into the box. The amount of electroactive FNR loaded on the electrode was estimated, as described previously, by measuring the area of the background-subtracted peaks observed in the absence of NADP^+^.^24^ The other enzymes are not electroactive, precluding such measurement. We thus adopted a strategy used previously, in which the ratio of enzymes in the loading solution was varied to optimize the electrocatalytic response. The concentration and retention of NADP^+^ in the electrode pores allow for the use of concentrations typically in the range of 5–20 μM.^26,27,30,31^ In this work, we used 20 μM NADP^+^ to optimize the signal to noise. This concentration was chosen based on a double titration experiment (both ATP and NADP^+^) for the coupling of CAR to FNR (Supporting Information 2.1).

For the experiments shown in Figures 2 and 3, the amount of FNR added to the loading solution was kept constant at 1.8 nmol, resulting in a concentration of 0.6 μM for Figures 2A and 0.5 μM for Figures 2B and 3A–D. With the exception of Figure 2A, the amount of CAR added to the loading solution was kept constant at 5.4 nmol, resulting in a loading concentration of 1.5 μM (4.3 nmol for Figure 2A resulting in a concentration of 1.4 μM). Consequently, the FNR/CAR ratio was 1:3 for each experiment (apart from 1:2.4 for Figure 2A). The AK/PK ratios were calculated in terms of the number of moles based on tetrameric PK and monomeric AK, where 1 (unity) represents 0.04 nmol of enzyme in the loading solution and 10 represents 0.4 nmol.

The reaction buffer for all experiments contained 10 mM cinnamic acid, 10 mM MgCl_2_, 10 mM KCl, 5 mM phosphate (KP_i_), and 100 mM HEPES, pH 7.5, referred to throughout as “standard reaction buffer” (Supporting Information 1.2.3 for details). The concentrations of AMP, PEP and, when included, ATP are listed in all figure legends and referred to in the main text. The NADP^+^ concentration in the bulk solution was constant throughout at 20 μM.

## Results

The first experiments were carried out to investigate how effectively the catalytic activity of CAR could be coupled to NADP(H) recycling by FNR without PK and AK also being loaded and present in the electrode nanopores: ATP was instead present in the external cell solution and was thus required to diffuse into the pores. Figure 2A shows a series of cyclic voltammograms recorded at a scan rate of 1 mV s^–1^ as the concentration of ATP was increased (the solution was mixed briefly at each addition of ATP). All other reactants were present in the solution from the start (i.e., 20 μM NADP^+^ in standard reaction buffer). The electroactive coverage of FNR determined before introducing NADP^+^ indicated a value of 22 pmol cm^–2^: as discussed previously, this value would equate to a concentration in the region of 0.2 mM throughout a 1 μm depth, but this ignores the space taken up by ITO itself, and the local concentration may be significantly higher. At the lower ATP concentrations (0.1–3.9 mM), no coupling was observed even after several cycles, although interestingly, both the reduction and oxidation peaks corresponding to the bidirectional catalysis of NADP^+^/NADPH interconversion by FNR were enhanced. This effect was reproducible in further experiments (Supporting Information 5.1). Although the origin of the ATP enhancement of FNR activity was not pursued further in this study (since it soon became clear that the NADPH regeneration stage, [electrons → FNR → NADPH], is not a bottleneck in the overall process), the observation confirmed that ATP has easy access into the electrode pores to reach the trapped enzymes.

A sigmoidal reduction wave (red scan), due to the catalytic recycling of NADPH back to FNR by CAR, eventually became evident after further additions of ATP resulted in a total concentration of 9.0 mM. The current continued to increase in subsequent successive scans without further ATP additions (pale gray scans graduating to black), stabilizing after 2.5 h (>15 cycles), whereupon an additional injection of ATP to give a total concentration of 18.8 mM did not result in any further increase.

A very different result was obtained if PK and AK were included in the loading solution (at a 1:10 AK/PK ratio, see Methods) with the aim of achieving in situ production and recycling of ATP (Figure 2B) instead of relying on using ATP as a solution-based single-use reactant. The cell solution contained 1 mM AMP along with a small quantity of ATP (10 μM) to act as a primer and 20 μM NADP^+^ (in standard reaction buffer), as for the experiment shown in Figure 2A. The electroactive coverage of FNR determined before the addition of NADP^+^ was comparable to the previous experiment, at approximately 27 pmol cm^–2^, despite the additional loading of the kinases in the porous electrode (see later for more information). The catalytic reaction was initiated by introducing PEP to give a final concentration of 2.5 mM. The violet scan corresponds to the background catalysis of NADP^+^/NADPH interconversion by FNR, and after initiation by PEP (red scan), the reductive current increased over 1.7 h (pale gray scans graduating to black). As before, a scan rate of 1 mV s^–1^ was used. Notable was the early appearance of a peak-type current response before a sigmoidal waveform was eventually established, indicating that ATP is initially consumed more rapidly than it can be replaced. The final current density obtained after 8 cycles (80 min) was similar in magnitude to that in Figure 2A, indicating that in situ generation of ATP by the kinase enzymes results in a comparable rate to that achieved using 8.8 mM ATP in the bulk solution.

Having established the efficiency of in situ ATP recycling, experiments were carried out to investigate the effect of varying the amounts and ratios of the two kinases: here, we emphasize again that only electroactive FNR can be quantified inside the electrode, so variations were performed by keeping the quantities of FNR and CAR constant, while changing the concentrations of PK and AK in the loading solution. Importantly, instead of including a low level of ATP as a primer, we exploited only the trace level of ATP contaminant present in commercial preparations of AMP.^53^ To detect and quantify this trace ATP in the AMP preparation, ^31^P NMR spectroscopy was carried out on a solution containing 20 mM AMP, and for comparison, a separate solution containing 20 mM AMP and 20 mM ATP. There were no detectable peaks corresponding to ATP in the spectrum for the 20 mM AMP sample (Supporting Information 3.1); therefore, the trace level of ATP must lie below the detection limit, equating in this case to <1% and thus <10 μM in 1 mM AMP. In all subsequent experiments, AMP was present in solution at 1 mM with the exception of Figure 4A (5 mM).

Figure 3 shows four sets of cyclic voltammograms (Panels A–D) in which the performance of the cascade was measured as a function of the amounts of AK and PK present in the loading solution. A scan rate of just 1 mV s^–1^ was used, so each cycle takes approximately 10 min. For each experiment, the electroactive coverage of FNR was determined before the addition of NADP^+^ and for AK/PK ratios of 1:1, 1:10, 10:1, and 10:10, and the values (in pmol cm^–2^) were 24.4 27.5, 10.7, and 20.5, respectively. There is no trend in FNR coverage with increasing amount of kinase since the values for each extreme (1:1 and 10:10) are comparable: the result for the 1:10 experiment is an outlier and shows that even with this lower amount of FNR present, the system is not FNR-limited since the current density is similar to the others, and a sigmoidal shape is retained. As before, initial cyclic voltammograms in each panel (violet) correspond to the interconversion of NADP^+^/NADPH catalyzed by FNR. Activation of the FNR/CAR/AK/PK cascade was initiated by adding PEP to a final concentration of 2.5 mM, all other reactants being present from the start (standard reaction buffer). In each experiment, the catalytic current increased with successive continuous cycles (shown in gray) until a maximum current was reached (black scan). The maximum current densities achieved in each case varied by only a factor of two, but the time taken to reach the maximum level decreased in the order: 1 AK:1 PK (3.7 h); 1 AK:10 PK (2 h); 10 AK:1 PK (40 min) and 10 PK:10 AK (20 min). These results—summarized in Panel E and particularly highlighted by comparing Panels B (1 AK:10 PK) and C (10 AK:1 PK)—show that although in situ ATP recycling requires both PK and AK, it is AK that is more important for shortening the lag phase and time required to reach an optimal steady state. The significance of this observation is discussed later. Furthermore, by comparing Figures 2B and 3B (both 1 AK:10 PK), it was established that the same result can be obtained without adding a known quantity of ATP as a primer, relying only on the amount present as a contaminant in AMP. Similar final current densities were reached at 100 and 120 min, respectively.

Figure 4 shows the results of two larger-scale chronoamperometry experiments for the synthesis of cinnamaldehyde, in which the enzyme cascade was driven at a fixed reducing potential of −0.42 V versus SHE. For both experiments, a “book” of five double-sided ITO@Ti foil electrodes (total surface area, 25 cm^2^) was used. After loading enzymes overnight, the electrodes underwent stringent rinsing in ultrapure water and were subsequently transferred to the glovebox in fresh buffer as described above.

In the experiment shown in Figure 4A, the electrode was loaded using the following mixture: 21.5 nmols of CAR, 9 nmols of FNR, 0.5 nmols of PK, and 1.7 nmols of AK (resulting in concentrations of 2.6, 1.1, 0.2, and 0.06 μM, respectively) in 100 mM TAPS pH 8. The reaction was initiated by the addition of NADP^+^ to a final concentration of 20 μM (all other reagents were present from the start (5 mM AMP and 5 mM PEP in standard reaction buffer); the cell solution (5 mL) was stirred with a magnetic flea. Injection of NADP^+^ resulted in a rapid increase in current (over the duration of 1–2 min, see inset), which decreased gradually to a low level after 12 h. A sample for NMR analysis was taken at approximately 15 h, after which the charge passed was equivalent to 1.29 × 10^–5^ mol—a concentration (in 5 mL) of 2.57 mM. Analysis of the NMR spectrum (Supporting Information 4.2) showed a concentration of 2.78 mM. The agreement between coulometric and NMR values is quite reasonable considering several factors associated with the small volume and current scales: (i) water evaporation over 15 h, which would concentrate the NMR sample (the cell was not perfectly sealed, and cinnamaldehyde is much less volatile than water); (ii) difficulty in allowing for background current (an estimation based on three background values gives 1.29 × 10^–5^ mol ± 0.25 × 10^–5^ mol, equivalent to a concentration of 2.78 mM ± 0.5 mM); (iii) migration of reactants and products into side arms. Since two moles of PEP should be consumed for each mole of cinnamaldehyde, the result demonstrates that the reaction can run until PEP is exhausted.

Figure 4B shows the results of a parallel experiment in which the cascade reaction was initiated instead by introducing a substoichiometric amount of PEP (1 mM, first green arrow) and then “refueled” twice by further additions during the time course. The electrode was loaded, as outlined in Figure 3A, with one change—a lower amount of PK was used (0.2 nmol). All other reagents were present from the start (20 μM NADP^+^ and 1 mM AMP in a standard reaction buffer). In contrast to the experiment initiated by NADP^+^, a lag of approximately 25 min was observed before the current started to increase (Figure 3B, inset). The current then started to decrease more rapidly than observed with the 5-fold higher PEP concentration. After a total of approximately 2.4 h, it was estimated, from the charge passed, that the PEP level should have decreased from 1 to 0.38 mM; at this point, the cascade was refueled (second green arrow) by adding a second equivalent addition of PEP (taking into account the PEP depleted during the first stage, this gave a total concentration in the bulk solution of ∼1.38 mM). In contrast to the injection of PEP made initially, no delay was observed, and the current increased immediately. After approximately 4.3 h, a sample was removed for NMR quantification of cinnamaldehyde (blue arrow), the result showing a concentration of 0.65 mM, equivalent to 3.28 μmol in the cell volume at that point, 5.05 mL (Supporting Information 4.3). For comparison, the total charge passed up to this point was 0.59 C, equating to 3.1 μmol cinnamaldehyde and a concentration of 0.60 mM. The cascade was refueled again at 4.4 h (third green arrow) by adding PEP to a give a final concentration of ∼5.15 mM [taking into account the amount of PEP estimated (through the charge passed) to have already been consumed at this stage and the volume change due to removal of the sample for NMR]. Once again, the increase in current was immediate. The current was monitored for a further 7 h. The total charge passed during the entire experiment was 1.28 C, equating to 6.6 μmol of cinnamaldehyde. The final concentration of cinnamaldehyde from NMR analysis (Supporting Information 4.3) in ∼4.55 mL of the total remaining solution was 1.79 mM, thus equating to approximately 8.1 μmol. Given the complications mentioned above, there is a good agreement between coulometric and NMR values.

## Discussion

The strong catalytic current observed in CVs and controlled potential experiments is directly related to the rate at which cinnamic acid is converted to cinnamaldehyde. As outlined in the Introduction, the results can be interpreted using basic electrochemical guidelines, and they reveal considerable insights into how and why nanoconfinement of an enzyme cascade produces such efficiency. All voltammetric waves attributed to cinnamic acid reduction have onset potentials that coincide closely with the reduction of NADP^+^ to NADPH. Furthermore, the waveforms are either sigmoidal or peak-type, demonstrating that interfacial electron transfer between ITO and FNR is not rate-limiting; had this been the case, the current would continue to increase with potential (rather than reach a plateau) as the rate of electrocatalytic regeneration of NADPH struggles to match demand by CAR. The clear implication is that the rate of cinnamic acid reduction is limited by subsequent chemical steps, either the turnover by CAR or the supply of ATP and reactants.

Attention focuses next on the striking contrast between the two experiments shown in Figure 2, that is, “bulk” ATP and “in situ” ATP. The first important point is that the final outcomes are similar in terms of waveshapes and current density; however, a very high solution concentration of ATP was required to achieve the final sigmoidal response for the simple FNR + CAR cascade, whereas only a priming amount of ATP (10 μM) was required if PK and AK had also been loaded into the electrode. A second point is more subtle. During the slow build-up of activity in the bulk-ATP experiment, the voltammogram is always sigmoidal; in other words, a steady state pertains throughout. In contrast, during the build-up of activity in the in situ experiment which uses AK and PK along with a small priming quantity of ATP and PEP in solution, peak-type voltammograms are observed early in the experiment, showing that an essential component is being depleted. The eventual transformation to a sigmoidal waveform shows that this component cannot be PEP (which is consumed having transferred from high (2.5 mM) concentration in bulk); instead, it is certainly ATP, initially present only at low concentration and prone to depletion until a sufficiently high level has been established.

An initial priming quantity of ATP is essential as there is no mechanism whereby the AK/PK recycling system can produce it starting from AMP alone. The results shown in Figure 3 show that just a trace (contaminating) amount of ATP (estimated to be no more than 10 μM) from the 1 mM AMP present in solution is all that is required to prime the recycling process. The four CV experiments, conducted with different amounts and ratios of AK:PK, reveal two facts. First, the waveforms increase in magnitude and transform from peak-type to sigmoidal, the rate of development increasing with total kinase loading and the transitory peak-type phase disappearing at the highest total loading. Consequently, early on, the available trace amount of ATP is quickly depleted, but eventually its production rate is sufficient to sustain a steady state. Second, the final sigmoidal current densities vary by less than a factor of two from very low (1:1) to high (10:10) kinase loading ratios and the final, marginal optimization is achieved by a tenfold increase in PK concentration.

Why is such a high concentration of ATP needed to achieve catalysis by CAR when it is supplied as a stoichiometric reactant? One key piece of evidence is the sensitivity of NADP(H) recycling by FNR, alone, to the presence of ATP, a result that could be reproduced in numerous separate experiments. Although the molecular interpretation of this enhancement of FNR activity is yet to be investigated, the result confirms the rapid arrival of ATP in the immediate pore locality. Attention is thus directed instead to AMP, which appears from solution kinetic studies to be a weak inhibitor of CAR (*K*_i_ = 8.2 mM).^54^ However, any such simple interpretation contradicts an important direct measurement, namely, that the bound AMP that is identified in the crystal structure of CAR was not introduced separately but remained throughout purification.^40^ The logical explanation for this discrepancy is that in the absence of turnover conditions, CAR adopts a resting inactive state in which AMP is tightly bound.

As illustrated in Figure 5, the tight binding of AMP in a resting state provides an explanation for why ATP introduced as a stoichiometric reagent is so ineffective compared to the localized recycling system. First, with regard to the lag period, in order to initiate the first catalytic cycle, AMP must first be released and sequestered. A negative effect of nanoconfinement would be to restrict its escape by diffusion into bulk solution, allowing it to rebind. In the absence of AK to sequester AMP, the catalytic activity, therefore, must rely on its escape through the pores. In contrast, when the entire AK/PK recycling system is in place, catalysis is initiated with just the trace amount of ATP that is present initially. The emphasis thus shifts to the fact that a crucial role of the recycling system is to remove AMP efficiently when nanoconfinement would retard its escape, an advantage that continues in subsequent catalytic cycles. The conclusion we reach is that local recycling not only supplies ATP but removes AMP, a function that is not performed when ATP is supplied as a stoichiometric reagent. The use of a two-enzyme ATP recycling branch as opposed to a single-enzyme system (e.g., a polyphosphate kinase) allows the roles of each stage to be dissected. The mechanism of CAR is thus more complicated than previously thought. The problem for precise modeling of the nanoconfined system lies in the uncertainty surrounding the actual quantities of CAR, AK, and PK actually present in the ITO pores.

The concept thus emerging also hinges on the advantage afforded by the fact that CAR is inherently the least active of the four enzymes. Were it to be highly active, then even once the activity has started to increase, the ATP required to prime the AK/PK service cycle would be spent (by CAR) before it could be invested (by AK) to produce ADP and produce (by PK) more ATP. Locally generated ATP is clearly much more effective than relying on its diffusion from solution. The results of the controlled potential electrolysis (chronoamperometric) experiments shown in Figure 4 provide firm support for these cascade dynamics. Provided the PK/AK service cycle has already been primed after introducing PEP, reduction of cinnamic acid to cinnamaldehyde starts rapidly after injecting NADP^+^. In contrast, if NADP^+^ is already present and the reaction is initiated instead by injecting PEP, there is a delay (Figure 4B inset).

Ultimately, the performance of the cascade depends on all enzymes and recyclable components being resident in the electrode. Given an optimal balance of all four enzymes, the catalytic rate is simply determined by the supply of PEP. The likelihood that CAR is the slowest enzyme allows us to place a lower limit on the local concentration of this enzyme that is active: based on a current density of 7 μA cm^–2^ and the published turnover frequency of 5 s^–1^, the surface (2D) coverage must be at least 7 pmol cm^–2^. Distributed across a depth of 1 μm (which ignores space taken up by ITO itself), the local concentration would need to be considerably higher than 0.07 mM.

The e-Leaf thus emerges as a confocal dual cofactor recycler to drive a complex cascade simultaneously by two sources of energy, electrical through FNR and NADP(H) and chemical via PEP, which serves as the fuel. In the light of recent work demonstrating that ITO electrodes can be scaled up to many hundreds of cm^2^ for small-scale production, it is clear that the e-Leaf can be developed to exploit the immobilization and nanoconfinement of entire enzyme-based synthetic pathways, avoiding steps and minimizing losses. The experiments described here were not carried out with the objective of optimizing turnover numbers for NADPH and ATP, which are, at best, just 90 for NADPH and >180 for ATP (from Figure 4A); there is plenty of scope for improvement in these metrics, longer experiments or lower concentrations being obvious ways forward. In any case, as these cofactors are being recycled locally, within the ITO pores, the real turnover numbers (per entrapped molecule) must be orders of magnitude higher. With the introduction of nanoconfined ATP recycling, an obvious exploitation of the discovery would be in the area of cancer research, where malfunctioning kinase enzymes are central.^55^ Finally, the ability to drive and study an enzyme that uses both reducing energy and ATP has further implications. In the case of CAR, simultaneous ATP consumption is required to increase the reducing power of NADPH, which is otherwise thermodynamically incapable of reducing a carboxylic acid to its aldehyde. Another enzyme of considerable current interest is nitrogenase, which uses electrons from a FeS protein and ATP, again in a coupled simultaneous process, to produce NH_3_ from N_2_.^56^ The lessons learnt from this investigation and the practical ways in which electrochemical and chemical energy can be simultaneously supplied to complex immobilized enzyme systems may, therefore, be harnessed to advantage in several different ways.

## Supporting Information Available

Enzyme production and electrochemical measurements; double ATP and NADP^+^ titration; NMR spectra; and effect of ATP on FNR catalysis (PDF)
