# Supplementary material for: A Nanoconfined Four-Enzyme Cascade Simultaneously Driven by Electrical and Chemical Energy, with Built-in Rapid, Confocal Recycling of NADP(H) and ATP
Source: ACS Catal. 2022 Jul 8;12(15):8811–21. doi: 10.1021/acscatal.2c00999 (PMC9361290; doi:10.1021/acscatal.2c00999)
Supplement: Supplementary file 1 — cs2c00999_si_001.pdf [file cs2c00999_si_001.pdf]

SUPPORTING INFORMATION

A nanoconfined four-enzyme cascade simultaneously driven by electrical and chemical energy, with built-in rapid, *confocal* recycling of NADP(H) and ATP

Clare F. Megarity,[1†*], Thomas R., I. Weald,[1] Rachel S. Heath[2], Nicholas J. Turner[2] and Fraser A. Armstrong[1*]

[1]Department of Chemistry, University of Oxford, South Parks Road, Oxford, OX1 3QR, UK

[2]School of Chemistry, University of Manchester, Manchester Institute of Biotechnology,

131 Princess Street, Manchester M1 7DN, UK

[†]Current address: School of Chemistry, University of Manchester, Manchester Institute of Biotechnology,

131 Princess Street, Manchester M1 7DN, UK

* Fraser A Armstrong
fraser.armstrong@chem.ox.ac.uk
* Clare F. Megarity
Clare.megarity@manchester.ac.uk

Contents

SI Section 1: Methods and Materials 1

1.1 Enzyme Production 1

1.2 Electrochemistry methods 2

1.2.1 Fabrication of ITO electrodes by electrophoretic deposition 2

1.2.2 Electrochemical Measurements 2

1.2.3 Standard Reaction Buffer and Reactant solutions 3

1.2.4 ^{1}H NMR Analysis 3

1.2.5 Enzyme loading 3

SI Section 2: Titration of ATP and $NADP^+$ 4

2.1 Double titration experiment (ATP and $NADP^+$) for the coupling of CAR to FNR in absence of the kinase pair 4

SI Section 3: ^{31}P NMR Spectra 5

3.1 ^{31}P NMR Spectra for AMP and ATP 5

SI Section 4: ^{1}H NMR Spectra relating to Figure 4 6

4.1 ^{1}H NMR spectrum for standard solutions of cinnamic acid and cinnamaldehyde 6

4.2 ^{1}H NMR standard curve and spectrum for the quantification of cinnamaldehyde produced in Figure 4A 7

4.3 ^{1}H NMR standard curve and spectrum for the quantification of cinnamaldehyde produced in Figure 4B 8

SI Section 5: The effect of ATP on the interconversion of NADP+/NADPH catalysed by FNR 9
5.1: FNR catalysis monitored in the presence of increasing concentrations of ATP 9
References 10

SI Section 1: Methods and Materials

1.1 Enzyme Production

FNR Ferredoxin $NADP^+$ Reductase was produced as described previously.[1-2] In brief: A transformation of *Escherichia coli* cells (BL21(DE3)) was carried out by uptake of a vector (aLICator pLATE 51, Thermo Scientific) containing the gene which encodes FNR from *Chlamydomonas reinhardtii* with an N-terminal His-tag and the genes to confer ampicillin resistance. The cells were then plated on lysogeny broth (LB)-agar plates containing ampicillin at 0.3 mM and incubated overnight at 37 °C. Resistance to ampicillin was used to select positive transformants. A single colony was used to inoculate 100 mL of LB media (25 gL^{-1}) containing ampicillin (100 μgmL^{-1}) and grown overnight at 37 °C in a shaking incubator (Innova) at 200 rpm. The following day, 10 mL of this overnight culture was diluted into 500 mL of fresh LB containing ampicillin at 0.3 mM and grown at 37 °C, 200 rpm for approximately 3 hours at which point they were induced by the addition of IPTG to a final concentration of 1 mM and grown under the same conditions for a further 3-4 hours. The cells were harvested by centrifugation and the pellets resuspended in cold buffer (50 mM HEPES, 150 mM NaCl, 10% V/V glycerol, pH 7.4) and stored at –80 °C until purification.

FNR purification: cells were lysed three times using a French press at 20 psi after which, cell debris was removed by centrifugation at 45000 rpm for 1 hour (Beckman Ultracentrifuge). The supernatant was loaded onto a Ni^{2+} HisTrap affinity column (GE Healthcare Life Sciences) using an Äkta purification system with dual wavelength absorbance detector. The column was washed with 50 column volumes of buffer (50 mM HEPES, 500 mM NaCl, 1 mM dithiothreitol (DTT), pH 7.4. A linear concentration gradient of imidazole reaching a maximum of 250 mM in ~30 min at a rate of 1.5 $mLmin^{-1}$, was used to elute 1 mL fractions. Those fractions containing FNR were based on the absorbance at 280 nm and 460 nm, the latter corresponding to the flavin cofactor. The fractions containing FNR can easily be detected by eye since they are bright yellow but attention to the peak at 280 is important for the identification of fractions at the beginning of the elution gradient which also may contain contaminant proteins. Fractions containing FNR were pooled and concentrated using a 10 KDa molecular weight cut-off centrifugal filter (Amicon®). Imidazole was removed by dialysis overnight at 4°C against 2 L of dialysis buffer (50 mM HEPES, 150 mM NaCl, 10% glycerol, 1 mM DTT, pH 7.4). FNR was aliquoted into small portions, (5 – 10 µL), flash frozen in liquid nitrogen and stored at –80 °C.

CAR CAR was produced similarly as previously described[3] with some changes. Two plasmids encoding a His-tagged CAR from *Segniliparus rugosus* (S*r)* and an untagged phosphopantetheine transferase *(Sfp) from Bacillus subtilis* were used to co-transform *Escherichia coli* cells (BL21(DE3)). The Sfp enzyme was required to carry out a post-translational modification of the co-expressed CAR (phosphopantetheinylation). The vector containing the gene to encode CAR also encoded the genes to confer resistance to kanamycin (kan) while the vector containing the gene to encode *Sfp*, also encoded resistance to streptomycin (strep); double resistance to both antibiotics was used to select positive transformants containing both plasmids. The cells were plated on a series of LB-agar plates containing three concentration sets for each antibiotic (plate A 0.03 $mgmL^{-1}$ kan and 0.05 $mgmL^{-1}$ strep; plate B 0.021 $mgmL^{-1}$ kan and 0.035 $mgmL^{-1}$ strep; plate C 0.015 $mgmL^{-1}$ kan and 0.025 $mgmL^{-1}$ strep). Positive transformants grew on all three plates and so the highest antibiotic concentration was chosen for subsequent expression. A single colony was used to inoculate 100 mL of LB containing 0.03 $mgmL^{-1}$ kan and 0.05 $mgmL^{-1}$ strep and the culture was grown overnight at 37 °C at 200 rpm; the overnight culture was then diluted (5 mL / 500 mL (X8)) into autoinduction

media (~35 gL^{-1}) (Formedium) containing 0.03 $mgmL^{-1}$ kan and 0.05 $mgmL^{-1}$ strep and grown for 72 hours at 20 °C (200 rpm). The cells were harvested by centrifugation, the pellets resuspended in cold buffer (50 mM HEPES, 150 mM NaCl, 10% V/V glycerol, pH 7.4) and stored at –80 °C until purification. The CAR was purified as described for FNR but with a less steep imidazole elution gradient reaching a maximum of 250 mM in ~60 min at a rate of 1.5 $mLmin^{-1}$ eluting in 1 mL fractions. The absorbance at 280 nm was used to identify the fractions containing CAR and this was confirmed by activity assay. UV-VIS spectroscopy was used to monitor the rate of NADPH depletion by CAR during its catalysis of the reduction of cinnamic acid to cinnamaldehyde. In brief, all reactants (200 µM NADPH, 10 mM cinnamic acid, 10 mM $MgCl_2$, 10 mM KCl, 10 mM ATP in 100 mM HEPES buffer pH 7.5) were added to a quartz cuvette and the absorbance at 340 nm measured for approximately 1 min before the addition of ~1-5 µL from each elution. A decrease in absorbance at 340 nm (due to the depletion of NADPH as it is oxidised to $NADP^+$ confirmed the presence of active CAR.

Active fractions were pooled and concentrated using a 50 KDa molecular weight cut-off centrifugal filter (Amicon®). Imidazole was removed by dialysis overnight at 4°C against 2 L of dialysis buffer (50 mM HEPES, 150 mM NaCl, 10% glycerol, 1 mM DTT, pH 7.4). CAR was aliquoted into 100 µL portions, flash frozen in liquid nitrogen and stored at –80 °C.

PK Pyruvate kinase (Type VII) from rabbit muscle was obtained from Merck (Sigma). CAS: 9001-59-6

AK Adenylate kinase from rabbit muscle was obtained from Merck (Sigma)

1.2 Electrochemistry methods

1.2.1 Fabrication of ITO electrodes by electrophoretic deposition

Indium tin oxide (ITO) electrodes were made by electrophoretic deposition of ITO particles (Sigma) onto titanium foil (Sigma), which acted as a conductive support. A suspension of ITO (1 $mgmL^{-1}$) and iodine (0.5 $mgmL^{-1}$) was prepared in acetone and sonicated for at least 30-45 min. The Ti foil electrode was aligned to face an auxiliary electrode of similar size (typically ITO glass or another piece of Ti foil) with approximately 1-2 cm separation. Both were held in this orientation in the ITO suspension and a voltage of 10 V was applied for approximately 7 minutes per electrode side. The ITO electrode was removed and allowed to dry in air before use.

For the large-scale chronoamperometry experiments shown in Figure 3, five ITO@Ti foil electrodes were made into a booklet by punching a hole through the top of each foil and threading Ti wire through several times to ensure electrical connection between each. This edge of the booklet was compressed using a vice to give stability and also to ensure all electrodes were connected to the wire (Figure S1). Before use, all electrodes were rinsed thoroughly in ultrapure water (Milli-Q™, 18 MΩcm).

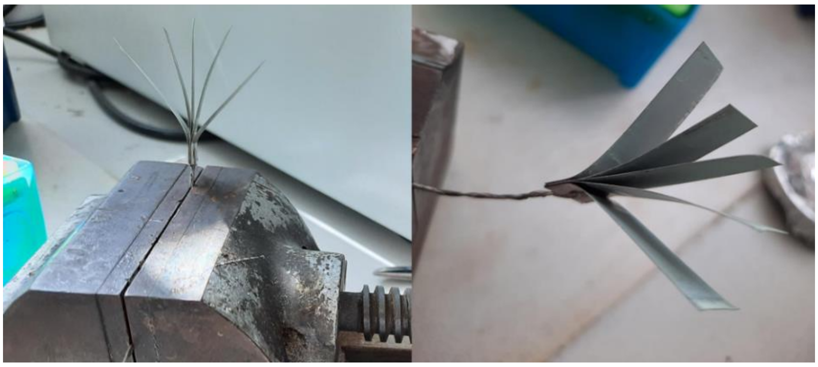

Figure S1: Construction of the ITO@Ti foil 'booklets' used in the large-scale chronoamperometry experiments shown in Figure 3.

1.2.2 Electrochemical Measurements

All experiments were performed under anerobic conditions using a glovebox (MBraun for all cyclic voltammetry or GBT Technologies for chronoamperometry) containing an N_2 atmosphere (O_2 < 2 ppm). Cyclic

voltammetry measurements were made using an Autolab potentiostat (PGSTAT128N) with Nova software; the potential was swept linearly between the upper and lower limits at a scan rate of 1 mVs^{-1} beginning at the more oxidising potential. Chronoamperometry experiments were controlled using an EcoChemie autolab with Nova software.

Measurements were made using either a two-compartment or three-compartment glass cell for cyclic voltammetry experiments and large-scale synthesis chronoamperometry experiments respectively. The two-compartment cell consisted of a working electrode compartment (water-jacketed and plumbed to a circulating water bath to maintain a constant temperature of 25 °C) connected, via a Luggin capillary, to a nonisothermal side arm housing a standard calomel (SCE) reference electrode. The working electrode compartment also contained a platinum wire counter electrode which completed the three-electrode configuration. The three-compartment cell was configured for an Ag/AgCl reference electrode and its additional side arm housed the counter platinum mesh electrode and was separated from the main working compartment by a glass frit. The side arms in both configurations contained 0.1 M NaCl.

Potentials (E) are quoted with respect to the standard hydrogen electrode (SHE) using the correction $E_{SHE} = E_{SCE} + 0.241$ V at 25 °C or $E_{SHE} = E_{Ag/AgCl} + 0.21$ V.[4]

1.2.3 Standard Reaction Buffer and Reactant solutions

For all experiments, the standard reaction buffer solution contained: 10 mM cinnamic acid, 10 mM $MgCl_2$, 10 mM KCl , 5 mM phosphate (K^+) and 100 mM HEPES (pH 7.5). Pyruvate kinase requires K^+ ions[5-6] and is highly selective for K^+ over Na^+[7-8] therefore KCl was included in the reaction buffer and the solution pH was adjusted using KOH rather than NaOH. Inorganic phosphate was included based on its enhancement of PK activity.[9] CAR and both kinase enzymes require Mg^{2+} ions, therefore $MgCl_2$ was included at 10 mM. A pH of 7.5 was chosen based on the activity of CAR previously measured in solution.[10]

For all additions of reactants to initiate or titrate, concentrated stocks were prepared in standard reaction buffer (for the PEP concentrated stock solution, cinnamic acid was omitted because a precipitate formed in this case) and the pH adjusted again to 7.5 so that the additions (typically 10 – 50 µL) to the cell solution did not dilute the other reactants or change the pH. Fresh concentrated stock solutions of $NADP^+$, PEP and AMP were prepared for each experiment. For the ATP titration experiment in Figure 1A, a 0.5 M stock ATP solution (dissolved in standard reaction buffer) was prepared and the pH adjusted to 7.5 so that the additions throughout the experiment did not alter the pH (the stock was flash frozen as single-use aliquots and stored at –80 °C). A concentrated stock solution of phosphate buffer (prepared by titrating the monobasic salt with dibasic salt to pH 7.5) was used as a stock solution such that a small volume addition would achieve the 5 mM final concentration in the cell solution.

The concentration of $NADP^+$ used throughout was constant at 20 µM; AMP was 1 mM with the exception of the experiment shown in Figure 3A which contained 5 mM AMP; PEP was 2.5 mM with the exception of Figure 3A which contained 5 mM and Figure 3B in which it was initially added at 1mM and replenished during the experiment.

1.2.4 ^{1}H NMR Analysis

A Bruker AVIIIHD 400 instrument was used to measure ^{1}H NMR spectra using a water suppression program. For the standard curve samples, cinnamaldehyde was dissolved in reaction buffer with 10% D_2O (D_2O was also added to experiment samples at 10%).

1.2.5 Enzyme loading

The desired number of moles of each enzyme were added to a buffer solution (100 mM TAPS pH 8) in a glass vial (a sufficient volume was used to cover the submerged electrode). The vial was placed on a magnetic stirrer in a 4 °C cold-room where it was stirred overnight using a magnetic flea. All electrodes were rinsed thoroughly in a stream of ultrapure water for at least 1-2 minutes before use. The electrode was then submerged into fresh buffer containing no enzyme, for transport into the glovebox.

SI Section 2: Titration of ATP and $NADP^+$

2.1 Double titration experiment (ATP and $NADP^+$) for the coupling of CAR to FNR in absence of the kinase pair.

Figure S2 shows a cyclic voltammetry experiment in which the coupled activity between CAR and FNR was monitored first as a function of ATP concentration (0.1 – 10 mM) whilst holding the concentration of $NADP^+$ constant at 5 µM (Figure S2 pale blue to dark blue scans) and second as a function of $NADP^+$ (10 and 20 µM) at a constant ATP concentration of 10 mM. An ITO@Ti foil electrode was loaded as described in the main text: the electrode was submerged in a 7 mL solution of buffer (100 mM TAPS pH 8) containing 4.3 nmoles of CAR and 1.9 moles of FNR and stirred overnight at 4 °C. The electrode was thoroughly rinsed in ultrapure water before use. The catalysis by FNR was monitored first with 5 µM $NADP^+$ for two consecutive scans after which the first addition of ATP (final concentration of 0.1 mM) was made, and two consecutive scans measured. Coupling was not evident and so ATP was subsequently titrated with a further 4 additions (with two scans measured for each addition (10 minutes per scan; pale blue to dark blue) to a maximum of ~ 9 mM ATP. After this time, since the coupling was not strong, the effect of additional $NADP^+$ (total concentration now 9.2 µM) was measured (two consecutive scans shown in pink) and the improvement was significant. A further increase in $NADP^+$ concentration to a total of 18 µM showed further enhancement of the coupled current which grew to a maximum over 8 scans (80 minutes). Based on this experiment, a concentration of 20 µM $NADP^+$ was chosen for all experiments shown in the main manuscript.

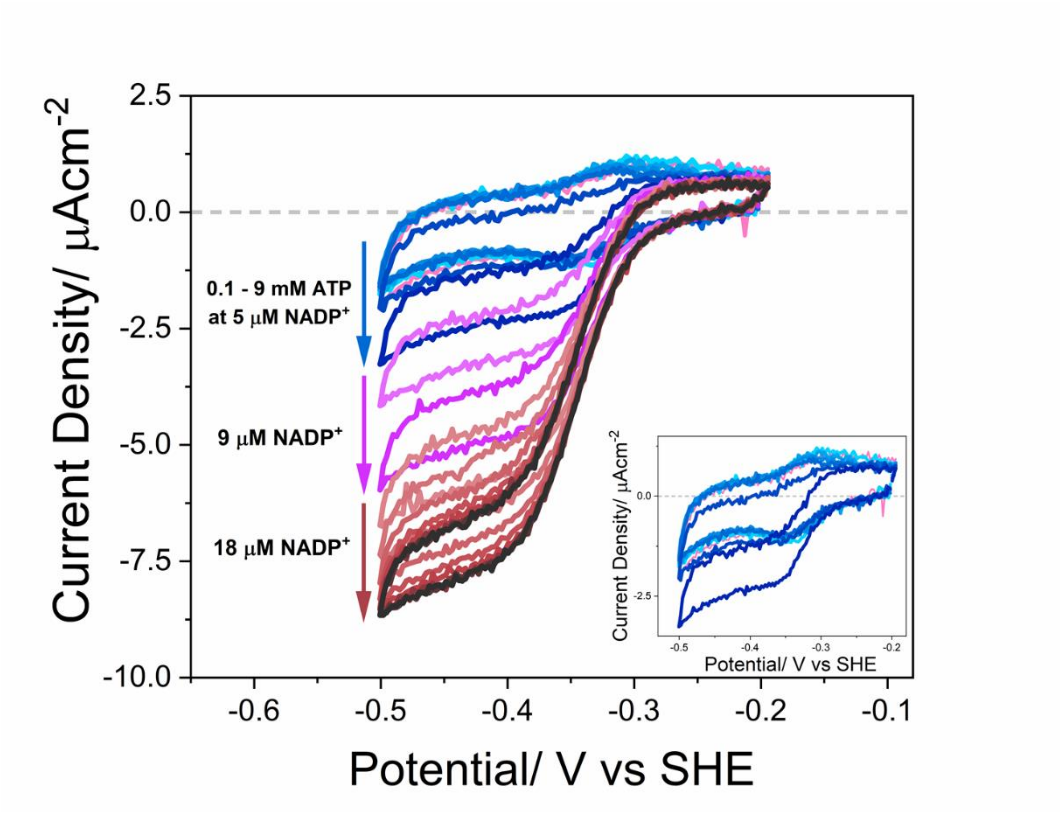

Figure S2. The reduction of cinnamic acid by CAR coupled to the interconversion of $NADP^+$/NADPH by FNR in the e-Leaf. Reactants, $NADP^+$ (5 µM), cinnamic acid (10 mM), $MgCl_2$ (10 mM) KCl (10 mM) in 100 mM HEPES buffer pH 7.5 present form the start; reaction initiated by the addition of ATP, titrated from 0.1 mM – 9 mM (pale blue to dark teal scans). Additional $NADP^+$ was added to a final concentration of 9 µM (pale pink scans). A further addition of $NADP^+$ to give a total concentration of 18 µM is also shown (medium pink graduating to dark purple). Other conditions: 25 °C, scan rate 1 mVs^{-1}, electrode stationary.

SI Section 3: ^{31}P NMR Spectra

3.1 ^{31}P NMR Spectra for AMP and ATP

Figure S4 shows the ^{31}P NMR spectra for two standard solutions containing 20 mM ATP and 20 mM AMP (A) and 20 mM AMP (B).

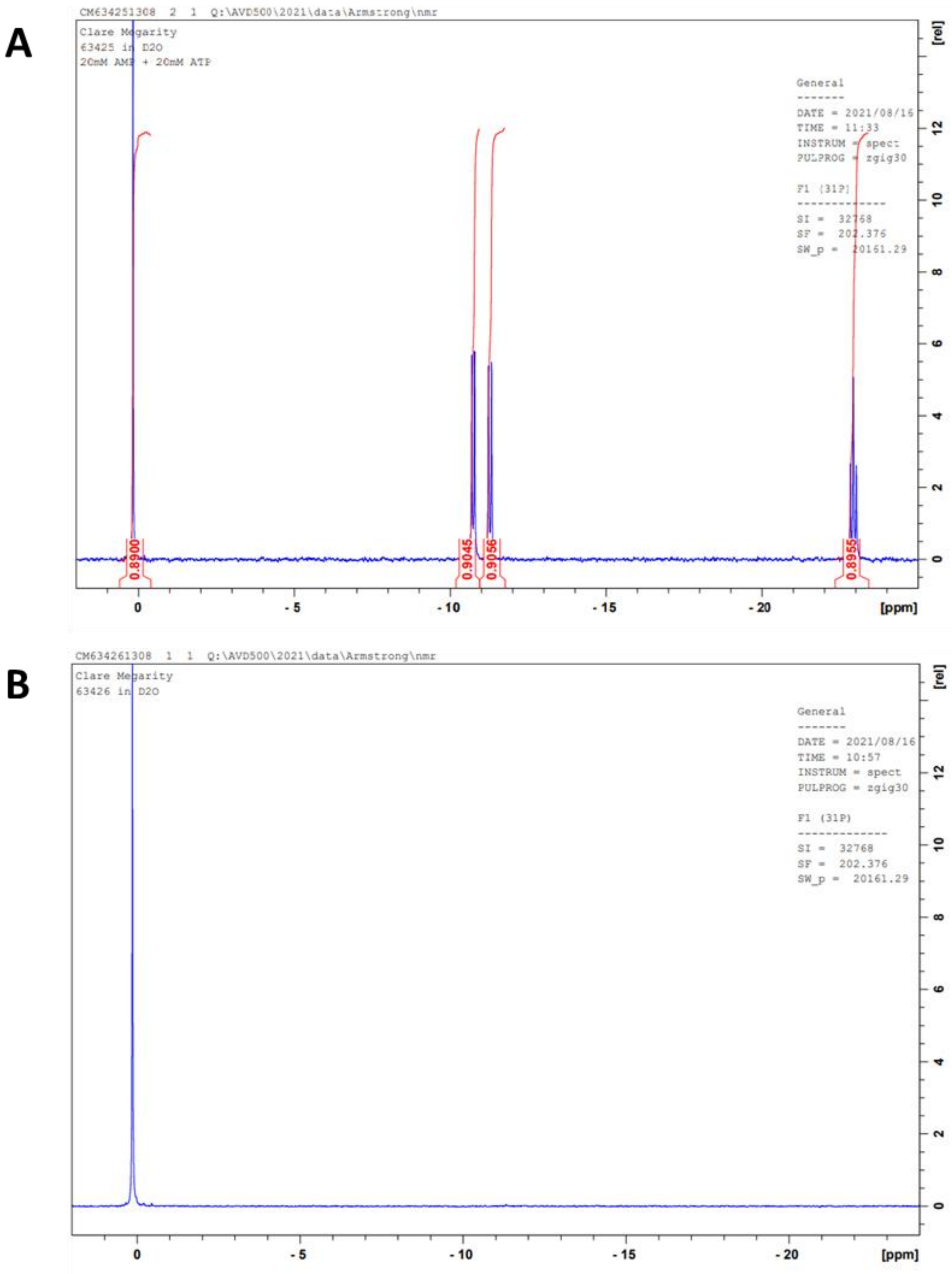

Figure S3 A: ^{31}P NMR spectrum for a solution containing 20 mM AMP and 20 mM ATP. **B:** ^{31}P NMR spectrum for a solution containing 20 mM AMP. Both were dissolved in 100% D_2O.

SI Section 4: ^{1}H NMR Spectra relating to Figure 4

4.1 ^{1}H NMR spectrum for standard solutions of cinnamic acid and cinnamaldehyde

Figure S4.1 shows the spectra for standard 10 mM solutions of cinnamic acid and cinnamaldehyde in the buffer used in all experiments (100 mM HEPES, 10 mM $MgCl_2$, 10 mM KCl, pH 7.5). A doublet peak at 9.5 ppm corresponding to the proton on the aldehyde group, was used for quantification in subsequent experiments.

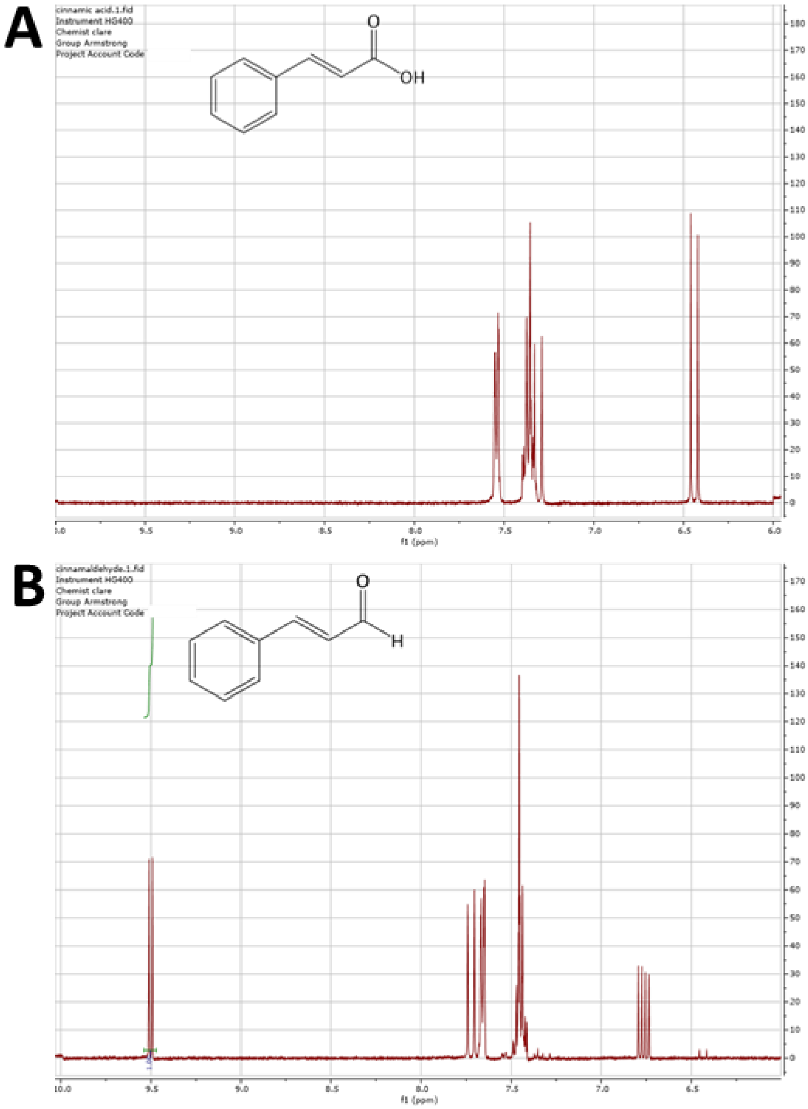

Figure S4.1 ^{1}H NMR spectrum for standard solutions of cinnamic acid and cinnamaldehyde. **A:** ^{1}H NMR spectrum for cinnamic acid (10 mM) in the reaction buffer (100 mM HEPES buffer pH 7.5 containing 10 mM $MgCl_2$, 10 mM KCl) with 10 % D_2O **B:** ^{1}H NMR spectrum for cinnamaldehyde (10 mM) in the same reaction buffer listed for A.

4.2 ^{1}H NMR standard curve and spectrum for the quantification of cinnamaldehyde produced in Figure 4A.

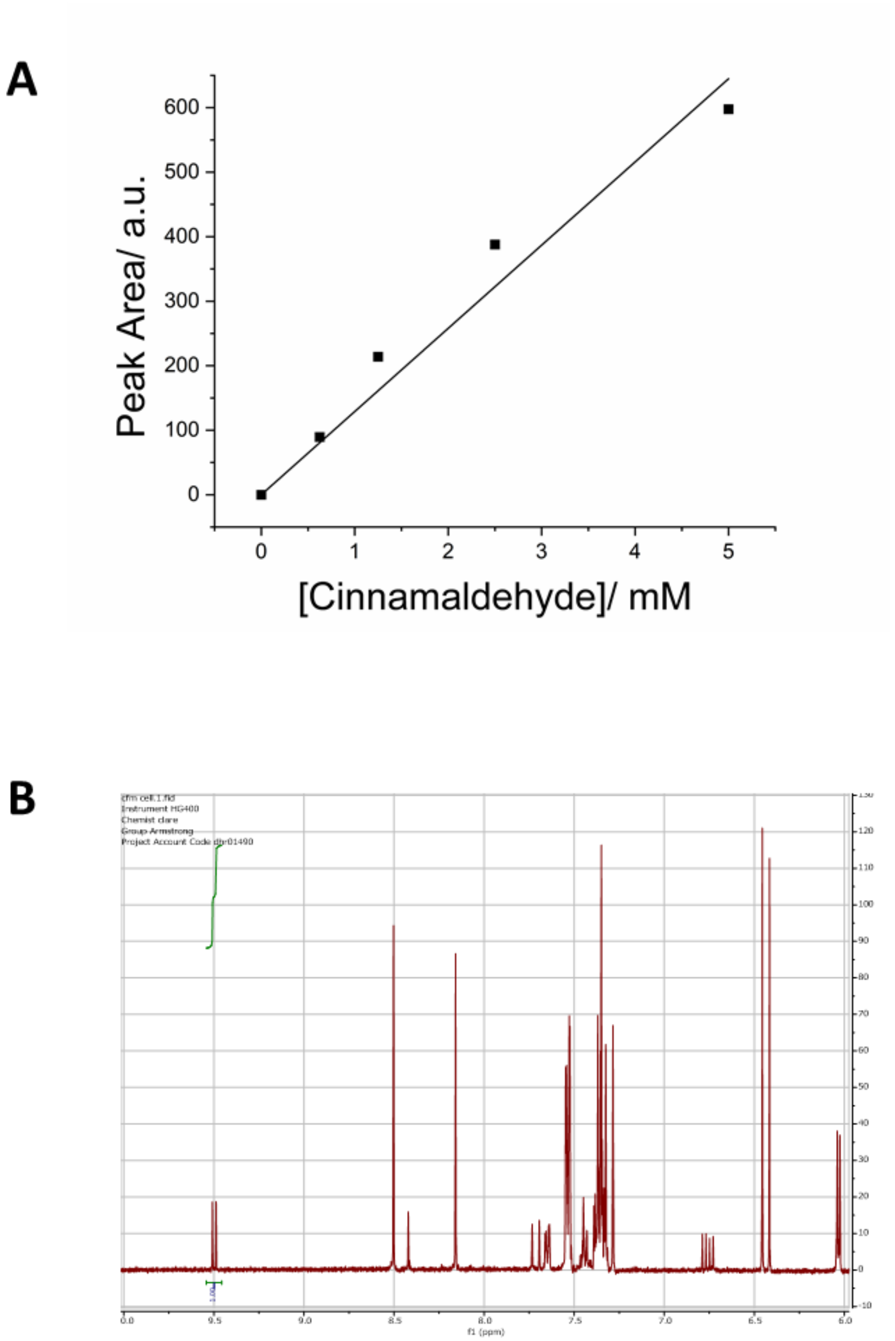

Figure S4.2 **A:** ^{1}H NMR standard curve for the quantification of cinnamaldehyde produced during the experiment in Figure 4A. **B:** ^{1}H NMR spectrum for the cell solution at the end of the experiment in Figure 4A.

4.3 ^{1}H NMR standard curve and spectrum for the quantification of cinnamaldehyde produced in Figure 4B.

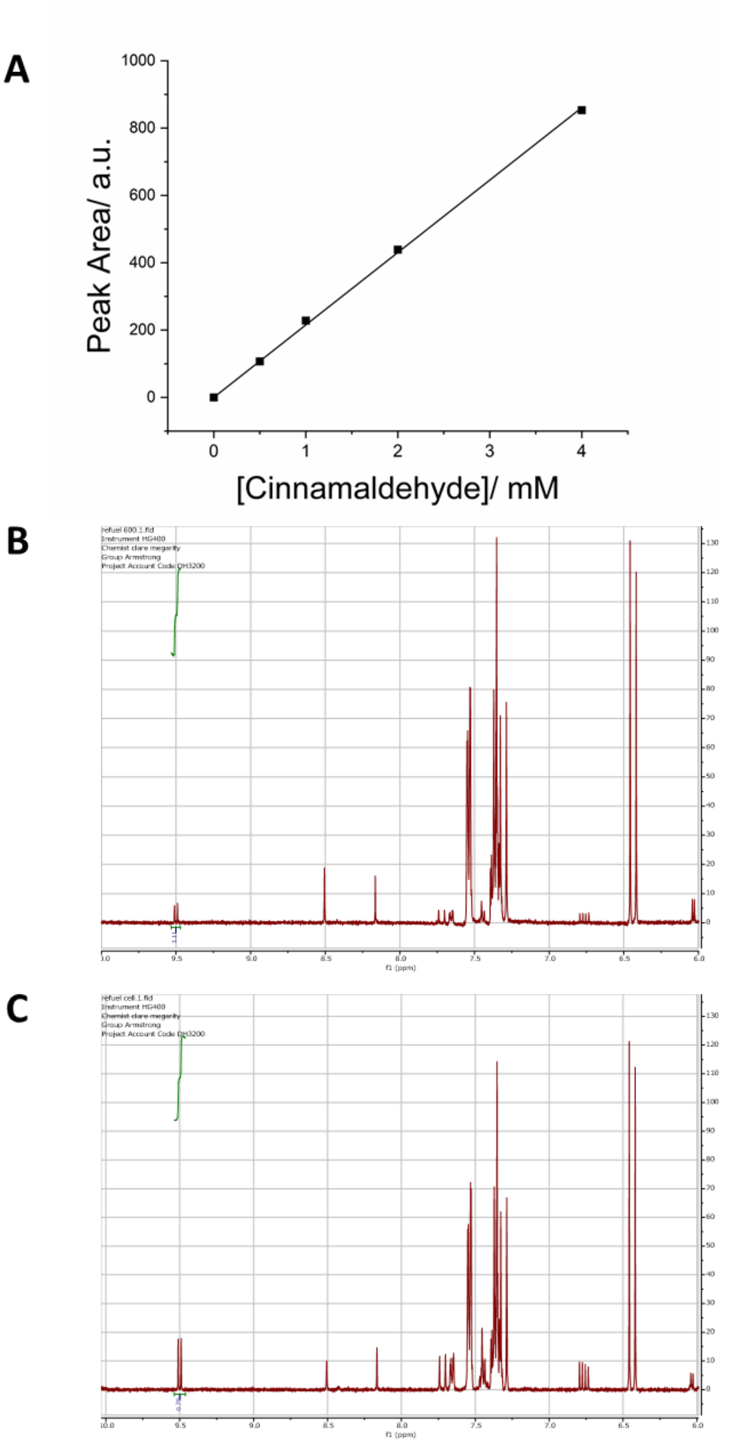

Figure S4.3 **A:** ^{1}H NMR standard curve for the quantification of cinnamaldehyde produced during the experiment in Figure 4B. **B:** ^{1}H NMR spectrum for the sample taken as indicated by the blue arrow **C:** ^{1}H NMR spectrum for the sample at the end of the experiment in Figure 4B.

SI Section 5: The effect of ATP on the interconversion of $NADP^+$/NADPH catalysed by FNR

5.1: FNR catalysis monitored in the presence of increasing concentrations of ATP

Figure S5 shows two experiments in which the effect of ATP on FNR catalysis was monitored by cyclic voltammetry. For the experiment shown in **A** an ITO@Ti foil electrode was loaded with FNR by stirring the electrode overnight in a 3 mL solution of TAPS buffer (100 mM pH 8) containing 1.8 nmoles of FNR and for **B** the electrode was loaded with both FNR (1.8 nmoles) and CAR (4.3 nmoles) in the same way. The scans for both experiments correspond to the initial catalysis of $NADP^+$/NADPH interconversion by FNR measured after a stable response had been established (pink scan) but before introducing ATP to the solution. During the subsequent cycles, ATP was titrated into the bulk solution (as very small aliquots to minimise dilution effects) from 0.1 mM (pale cyan) to 10 mM (dark blue).

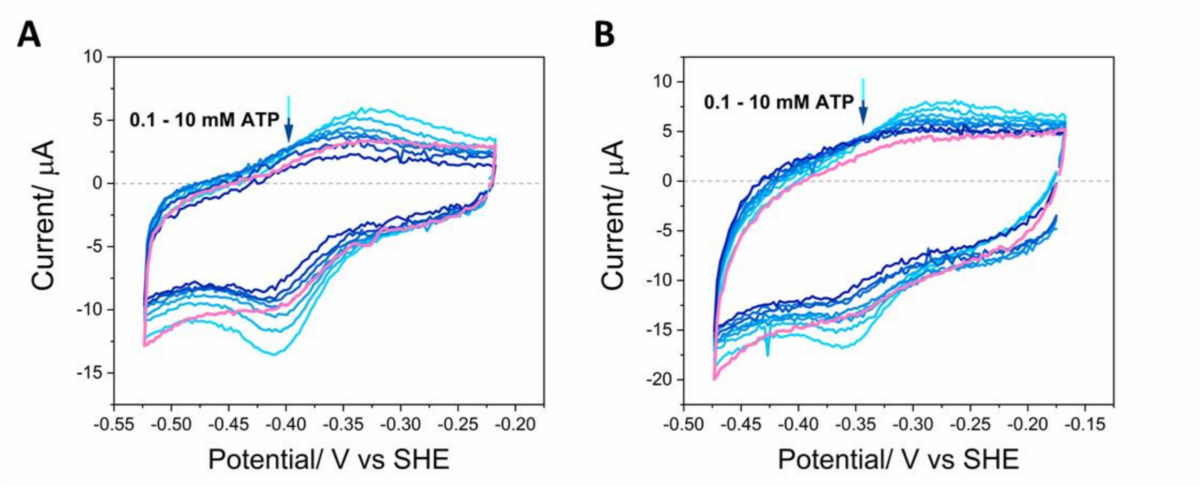

Figure S5. The effect of ATP on the interconversion of $NADP^+$/NADPH catalysed by FNR. **A:** The electrode is loaded with FNR only (one ITO@Ti foil electrode loaded overnight as for all other experiments in the main text; loading solution contained 1.8 nmoles of FNR, in 100 mM TAPS pH 8). Pink scan: the interconversion of $NADP^+$/NADPH (20 μM) by FNR. Blue scans: the titration of ATP from 0.1 mM (cyan) to 10 mM (dark blue). For consistency with experiments in the main text, all reactants present despite the absence of the kinases and CAR: cinnamic acid (10 mM), $MgCl_2$ (10 mM) KCl (10 mM) in 100 mM HEPES buffer pH 7.5. **B:** Similar experiment to that shown in **A** this time with CAR also loaded (4.3 nmoles in the loading solution) in addition to FNR (all reactants present as for **A**, except for cinnamic acid since this would have resulted in unwanted coupling to CAR and the purpose of this experiment was to monitor the effect of ATP on FNR catalysis in the presence of CAR but without its activity. Other conditions: 25 °C, scan rate 1 mVs^{-1}, electrode stationary.

References

1. Megarity, C. F.; Siritanaratkul, B.; Cheng, B.; Morello, G.; Wan, L.; Sills, A. J.; Heath, R. S.; Turner, N. J.; Armstrong, F. A., Electrified Nanoconfined Biocatalysis with Rapid Cofactor Recycling. *ChemCatChem* **2019,** *11* (23), 5662-5670.
2. Megarity, C. F.; Siritanaratkul, B.; Heath, R. S.; Wan, L.; Morello, G.; FitzPatrick, S. R.; Booth, R. L.; Sills, A. J.; Robertson, A. W.; Warner, J. H.; Turner, N. J.; Armstrong, F. A., Electrocatalytic Volleyball: Rapid Nanoconfined Nicotinamide Cycling for Organic Synthesis in Electrode Pores. *Angew. Chem* **2019,** *58* (15), 4948-4952.
3. Gahloth, D.; Dunstan, M. S.; Quaglia, D.; Klumbys, E.; Lockhart-Cairns, M. P.; Hill, A. M.; Derrington, S. R.; Scrutton, N. S.; Turner, N. J.; Leys, D., Structures of carboxylic acid reductase reveal domain dynamics underlying catalysis. *Nat Chem Biol* **2017,** *13* (9), 975-981.
4. Bard, A., J. and Faulkner, L., R., *Electrochemical Methods : Fundamentals and Applications*. 2 ed.; Wiley: Chichester, 1980.
5. Kachmar, J.; Boyer, P., Kinetic analysis of enzyme reactions II. The potassium activation and calcium inhibition of pyruvic phosphoferase. *Journal of Biological Chemistry* **1953,** *200* (2), 669-682.
6. Oria-Hernández, J.; Cabrera, N.; Pérez-Montfort, R.; Ramírez-Silva, L., Pyruvate kinase revisited: the activating effect of K+. *J Biol Chem* **2005,** *280* (45), 37924-9.
7. Ramírez-Silva, L.; Oria-Hernández, J., Selectivity of pyruvate kinase for Na+ and K+ in water/dimethylsulfoxide mixtures. *European Journal of Biochemistry* **2003,** *270* (11), 2377-2385.
8. Nowak, T.; Suelter, C., Pyruvate kinase: Activation by and catalytic role of the monovalent and divalent cations. *Molecular and Cellular Biochemistry* **1981,** *35* (2), 65-75.
9. Koster, J. F.; Hulsmann, W. C., The influence of inorganic phosphate and phosphorylated hexoses on the activity of pyruvate kinase. *Arch Biochem Biophys* **1970,** *141* (1), 98-101.
10. Gahloth, D.; Dunstan, M. S.; Quaglia, D.; Klumbys, E.; Lockhart-Cairns, M. P.; Hill, A. M.; Derrington, S. R.; Scrutton, N. S.; Turner, N. J.; Leys, D., Structures of carboxylic acid reductase reveal domain dynamics underlying catalysis. *Nature Chemical Biology* **2017,** *13* (9), 975-981.
